# Maternal health, pregnancy and birth outcomes for women involved in care proceedings in Wales: a linked data study

**DOI:** 10.1186/s12884-020-03370-4

**Published:** 2020-11-16

**Authors:** L. J. Griffiths, R. D. Johnson, K. Broadhurst, S. Bedston, L. Cusworth, B. Alrouh, D. V. Ford, A. John

**Affiliations:** 1 Population Data Science, Swansea University Medical School, Swansea, SA2 8PP UK; 2 Centre for Child & Family Justice Research, Lancaster University, Lancaster, LA1 4YW UK

**Keywords:** Care proceedings, Administrative data, Data linkage, Pregnancy, Birth outcomes, Mental health, Case-comparison study

## Abstract

**Background:**

Under the Children Act 1989, local authorities in Wales, UK, can issue care proceedings if they are concerned about the welfare of a child, which can lead to removal of a child from parents. For mothers at risk of child removal, timely intervention during pregnancy may avert the need for this and improve maternal/fetal health; however, little is known about this specific population during the antenatal period. The study examined maternity characteristics of mothers whose infants were subject to care proceedings, with the aim of informing preventative interventions targeted at high risk mothers.

**Methods:**

Anonymised administrative data from Cafcass Cymru, who provide child-focused advice and support for family court proceedings in Wales, were linked to population-based maternity and health records held within the Secure Anonymised Information Linkage Databank. Linked data were available for 1111 birth mothers of infants involved in care proceedings between 2015 and 2018. Findings were benchmarked with reference to an age-deprivation-matched comparison group (*n* = 23,414), not subject to care proceedings but accessing maternity services during this period. Demographic characteristics, maternal health, reproductive history, interaction with midwifery services, and pregnancy and birth outcomes were examined. Descriptive and statistical tests of independence were used.

**Results:**

Half of the women in the cohort (49.4%) resided in the most deprived areas. They were more likely to be younger at entry to motherhood (63.5% < 21 years-of-age compared to 42.7% in the comparison group), to have mental health (28.6% compared to 8.2%) and substance use issues (10.4% compared to 0.6%) and to smoke (62.7% compared to 24.8%) during pregnancy. The majority first engaged with maternity services within their first trimester of pregnancy (63.5% compared to 84.4%). Babies were more likely to be born preterm (14.2% compared to 6.7%) and, for full-term babies, to have low birthweights (8.0% compared to 2.8%).

**Conclusion:**

This novel linkage study highlights multiple vulnerabilities experienced by pregnant mothers who have experienced care proceedings concerning an infant. Policy and practice colleagues require a clearer picture of women’s needs if child protection and health services are to offer effective services which prevent the need for family court proceedings and infant removal.

## Background

Infants are entirely dependent on their caregivers for their safety and well-being. In cases where an infant is identified as being at risk of suffering significant harm from one or both parents, a local authority may seek to remove a child and issue care proceedings under Section 31 (s.31) of the 1989 Children Act (England and Wales). Although there is clear international consensus that all children must be protected from harm, there are escalating numbers of very young babies in care proceedings in England and Wales, with the incidence rate almost doubling from 2015 to 2018, when it reached 83 cases per 10,000 live births [1]. Whilst infants (aged less than 12 months old) comprise around 30% of all s.31 cases in Wales, more than half of these are newborns [1]. This is prompting searching questions about what more might be done during *pregnancy,* to reduce the need to enact care proceedings through the family justice system [2]. In England and Wales, the Children Act 1989 remains the authorising legal framework for practice [3–5] and aims to ensure practitioners strike an effective balance between family support and more intrusive child protection intervention, including issuing care proceedings in the family court. However, the over-arching conclusion of the recent Care Crisis Review [6], is that urgent investment in tailored preventative services is needed, if the rising tide of very young babies entering care is to be stemmed. It is this argument which has driven recent changes in prenatal reporting in a number of international jurisdictions [5, 7].

Antenatal services are currently designed to identify and address maternal vulnerabilities during pregnancy. However, given rising rates of newborn entry to care, there are concerns about the fit between how services as currently configured, and the *specific* needs of mothers who lose infants from their care. At present it is difficult to address these concerns directly, due to a dearth of empirical evidence focused specifically on the population of birth mothers in question and their pregnancies. Although there is a wealth of literature reporting an association between socio-economic status, mental health difficulties, substance use (alcohol and drugs) and pregnancy [8–10], this literature is insufficiently focused on the population of birth mothers in question. For example, the published literature offers no insights about the timing of this particular population of women’s engagement with ante-natal services, despite the fact that pregnancy provides an absolutely critical space for intensive work to address parental vulnerabilities associated with infant removal. Current gaps in evidence arguably, reflect the lack of integrated, interdisciplinary analysis of pressing questions about birth parents in care proceedings and/or limited opportunities for linking health and social care records [11].

This article sought to address these evidence gaps by providing a first descriptive picture of: a) maternal health and well-being prior to and during pregnancy, b) interaction with maternity services, and c) pregnancy and birth outcomes for women subject to care proceedings during their child’s first year of life in Wales. For the first time, the research team were able to utilise population-level administrative data collected routinely by Cafcass Cymru (a Welsh government organisation that represents children’s best interests in family justice proceedings in Wales) linked to maternity and electronic health records (EHRs), to produce an integrated retrospective picture of women’s maternity profiles.

## Methods

### Study design

This study used a population-level cohort study with a matched comparison group, with the group of interest being mothers involved in care proceedings regarding an infant. The comparison group were mothers who had given birth over the same time period, as described below, but had not been involved in care proceedings. Measures of interest covered four areas: i) demographic characteristics, ii) maternal health and well-being, iii) maternal reproductive history and interaction with midwifery services and, iv) immediate pregnancy and birth outcomes.

### Data sources and linkage

Data were obtained via the SAIL (Secure Anonymised Information Linkage) Databank [12–15], which contains extensive anonymised health and administrative data about the population of Wales, accessible in anonymised form via a secure data sharing platform, all underpinned by an innovative and proportionate Information Governance model. All data within the SAIL Databank are treated in accordance with the Data Protection Act 2018 and are compliant with the General Data Protection Regulation.

The primary source of family justice information was a routinely produced extract of administrative case management data maintained by Cafcass Cymru. At the time of study design, the SAIL Databank held all instances of s.31 care proceedings initiated between January 2011 and December 2018. Relevant case information for this study included: child’s week of birth and sex; adult respondent’s week of birth, sex, and indication of relationship to the child; the local authority making the application; and the date on which the s.31 application was submitted. Further detail on Cafcass Cymru data are available elsewhere [16–18].

The Maternity Indicator Dataset (MIDS) [19] captures data from local health board systems relating to women at their initial antenatal assessment, known as ‘booking’, and to mother and baby (or babies) at labour and birth. MIDS was established to enable the Welsh Government to monitor outcome indicators and performance measures, established to measure the effectiveness and quality of Welsh maternity services. At the time of study design, the SAIL Databank held information from April 2014 for initial assessment events, and from January 2015 for birth events, until December 2018.

Linkage was made to the Welsh Demographic Service Dataset (WDSD), which provides demographic characteristics of people registered with a general practice (GP) in Wales, providing residents’ demographic and address details.

The Patient Episode Database for Wales (PEDW) [20] contains data for all episodes of in-patient and day-case activity in NHS Wales hospitals, including elective and emergency admissions, minor and major operations and hospital stays for childbirth. Relevant case information for this study included: admission dates, admission type and ICD-10 [21] diagnosis codes for each episode of care, relating to the reason for admission and co-morbidities for each patient. The data for this study was restricted to admission dates from 2013 to 2018 inclusive.

The Welsh Longitudinal General Practice (WLGP) data contains GP records for patients registered with a Welsh GP, for approximately 80% of practices that supply data to the SAIL Databank. Each record contains information such as the event date and Read codes (a hierarchical coded thesaurus of clinical terms) which are used to record patient diagnoses and procedures. For this study, the event date coverage was restricted from 2013 to 2018 inclusive.

During the anonymisation process of data sources within the SAIL Databank, individuals are assigned an anonymised linking field (ALF) based on their National Health Service number, name, sex, date of birth and postcode. This anonymisation and linkage methodology has previously been described [12]. ALFs were used to link the datasets outlined above. Researchers did not have access to personal identifiable data.

### Study population

**Fig. 1:**
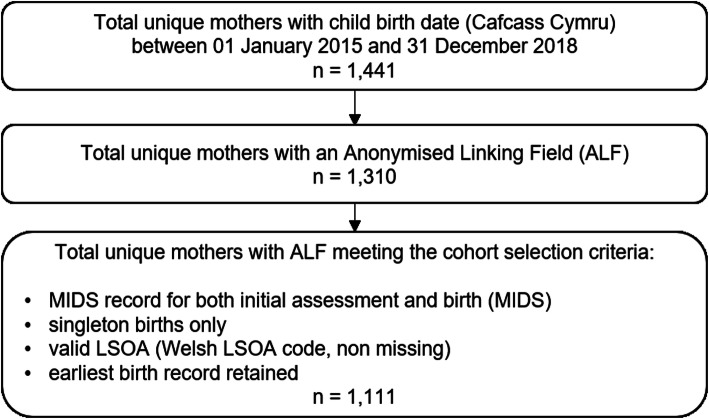
Flow diagram for creation of the study cohort

The mothers included in this study were birth mothers of infants, born between 1 January 2015 and 31 December 2018, who were involved in s.31 care proceedings in Wales during their first year of life (*n* = 1441). This timeframe was taken due to the availability of MIDS data in the SAIL Databank, as described above. Of these, 1,310 (90.9%) were assigned an ALF enabling linkage to the other data sources. The sample was further restricted to mothers with MIDS assessment and birth information, and only included singleton births to mothers aged between 12 and 59 with a valid Welsh LSOA recorded. For mothers giving birth multiple times within the study period only the first birth was included. The final cohort consisted of 1111 mothers (Fig. 1). Mothers excluded during this selection process were slightly older (*p* < 0.05) but did not vary on residential area of deprivation (*p* = 0.45).

Matched sampling was utilised to create a comparison group of mothers, selected from the wider population of mothers with available MIDS data within the SAIL Databank for the same time period. Mothers were matched on age band at the point of birth and deprivation quintile, with random selection used to restrict numbers in the comparison group which consisted of a total of 23,414 mothers.

### Measures

#### Maternal demographic characteristics, health and well-being

The Welsh Index of Multiple Deprivation (WIMD) is the Welsh government’s official deprivation measure for statistical geographies in Wales - Lower Layer Super Output Areas (LSOA). Each LSOA is made up of households within postal codes aggregated to reach a minimum number of people that satisfy statistical disclosure control requirements. For the 2011 census, from which LSOAs are derived, on average the population of a Welsh LSOA was 1614 [22]. WIMD is linked to the LSOA statistical geographies and is ranked from 1 (most deprived) to 1909 (least deprived), which was divided into deprivation quintiles for this study (1 - most deprived, to 5 - least deprived). LSOA was obtained from the MIDS or, where not present, the WDSD for each mother.

Two measures of maternal age were used, the first being mother’s age at birth of the child involved in the care proceedings (and categorised into age bands: < 21, 21–25, 26–30, 31–35, and > 35). The second was maternal age at entry to motherhood (had their first child), using the earliest EHR of childbirth, regardless of whether the child was involved in care proceedings.

Two measures of maternal mental health were examined. Firstly, women were defined as having a mental health condition if they self-reported (MIDS) having one or more of the following conditions at their initial maternity assessment: Puerperal psychosis (severe postnatal depression); Bipolar affective disorder/manic depression; Psychosis; Psychotic depression; Schizophrenia; or other mental health condition. Secondly, mothers’ primary care (WLGP) and hospital (PEDW) EHRs were analysed for the presence of clinical codes indicating mental health related contacts or admissions. If a mother had one or more mental health related contact or admission code recorded i) during pregnancy, and ii) within the 2 year period prior to the birth of the child involved in the care proceedings, they were categorised as having a mental health condition. Code lists were developed and provided by the Adolescent Mental Health Data Platform [23] and based on previously published work or in consultation with clinicians. We included codes for: common mental disorders e.g. depression and anxiety [24, 25]; severe mental illness [26]; eating disorders [27]; neurodevelopmental disorders e.g. Attention Deficit Hyperactivity Disorder, Autistic Spectrum Disorder; and Conduct Disorders.

Substance use can represent substance use or substance dependence, and was determined in this study through electronic searches of clinical codes used again within primary care (WLGP) and hospital (PEDW) EHRs, including codes for diagnoses, symptoms and medications indicative of problem, harmful or hazardous use of alcohol and/or illicit drugs. If a mother had any such recorded code i) during pregnancy, and ii) within the two-year period prior to the birth of the child involved in the care proceedings, they were classified as having a substance use contact or admission during this period. Code lists were developed with advice from clinicians including a substance use disorder nurse and also provided by the Adolescent Mental Health Data Platform [23]^.^

Valid maternal weight (30 kg to 250 kg) and height (1.2 to 2 m) records (available within MIDS), taken at initial assessment, or within the 10–12 week gestation period (when not undertaken at initial assessment) were used to calculate Body Mass Index (BMI) (kilogram/m^2^). Mothers were subsequently defined as being: underweight, < 18.5; healthy weight, 18.5–24.9; overweight, 25–29.9; obese, 30–39.9; or morbidly obese ≥40.

Smoking status (smoker vs non-smoker) was defined at initial maternity assessment, and birth, using self-report or validated via carbon monoxide testing.

Breastfeeding was captured in MIDS as intention to breastfeed the baby at birth, rather than actual breastfeeding initiation (when a mother begins to feed her infant milk from her breast).

#### Maternal reproductive history and interaction with midwifery services

Gravida was defined as the total number of pregnancies for a woman (including current pregnancy), regardless of whether a pregnancy was carried to term; this was categorised as: one, two, three, or four or more.

Parity was defined as the number of times the mother had given birth to a live neonate at 24 weeks or more, regardless of whether the child was viable or non-viable (i.e. still births). As this data was collected at initial assessment, this measure relates to their previous live births and did not include birth of current child involved in care proceedings. We created three categories: never previously given birth; previously given birth only once; and previously given birth more than once.

At initial assessment, gestation period (in weeks) was obtained by ultrasound scan or, where this was not undertaken, estimated from the first day of last menstrual period. This study examined the proportion of women who received their initial assessment within the first (conception to 12th week of pregnancy), second (13 to 27 weeks) or third (28 weeks until birth) trimester.

#### Immediate pregnancy and birth outcomes

These outcomes included place (hospital or non-hospital) and mode of birth: vaginal (unassisted), instrumental (ventouse or forceps delivery), or caesarean section (elective or emergency).

Gestational age at onset of labour was used as a proxy for gestation age at birth; this information was used to categorise babies born prematurely: preterm (< 37 weeks), full term (37 to 42 weeks), and post term (> 42 weeks).

Birthweight, recorded in grams at birth, was used to define low (less than 2500 g), ‘healthy’ (2500–3999 g) and high (> = 4000 g) birthweights.

Finally, Apgar scores (a new-born test involving numerous health checks (baby’s skin colour, heart rate, reflexes, muscle tone, and respiration) to assess if extra medical care or emergency care is needed) taken at 5 minutes were used, and categorised as less than 7 or 7 or more (indicating either an immediate unhealthy or healthy baby status at birth, respectively).

### Data analysis

Analyses were carried out through descriptive reporting and bivariate testing. We calculated the proportions of mothers or babies with characteristics of interest during pregnancy or at birth, or - in the case of mental health and substance use – during pregnancy or the 2 year-period prior to the birth of the child involved in family court proceedings. One-way analysis of variance tests were computed to compare the means between the cohort and matched comparison group for continuous variables (e.g. maternal age). Chi-squared analyses was used to investigate differences between these groups for all remaining variables. In advance of the analysis being performed, the significance-level for all testing was set at < 0.001. As a sensitivity analysis, unadjusted odds ratios with 99.9% Confidence Intervals were also calculated to confirm strength, direction and substantive meaning (supplementary material; findings not discussed given concurrence of results). Data processing and analyses were carried out using SQL and R [28].

## Results

### Maternal demographic characteristics

Half of the women in the cohort (49.4%) resided in the most deprived quintile, with 75.8% living in the two most deprived quintiles (Table 1). The mean age at current birth was 26.0 years (Table 1). One quarter of the mothers (24.8%) were under 21 years old when they gave birth, 74.8% were aged 30 or under, and 9.6% fell within the oldest age band, over 35 years. Given the age-deprivation matching process, the residential and age distribution for the comparison group was similar.

**Table 1:** Maternal demographic characteristics for the cohort (*n* = 1111) and comparison group (*n* = 23,414)

Variable	Level	Cohort n (%)	Comparison n (%)	***p***-value
Deprivation at childbirth	Quintile 1: Most Deprived	549 (49.4)	11,641 (49.7)	0.998
	Quintile 2	293 (26.4)	6186 (26.4)	
	Quintile 3	158 (14.2)	3317 (14.2)	
	Quintile 4	80 (7.2)	1633 (7.0)	
	Quintile 5: Least Deprived	31 (2.8)	637 (2.7)	
Age at current birth	< 21 years	276 (24.8)	5,830 (24.9)	1.0
	21 to 25 years	297 (26.7)	6281 (26.8)	
	26 to 30 years	259 (23.3)	5475 (23.4)	
	31 to 35 years	172 (15.5)	3601 (15.4)	
	> 35 years	107 (9.6)	2227 (9.5)	
Age at current birth (mean (SD))^a^		26.0 (6.5)	26.1 (6.2)	0.482
Age at entry to motherhood	< 21 years	699 (63.5)	9847 (42.7)	< 0.001
	21 to 25 years	245 (22.3)	6520 (28.3)	
	26 to 30 years	82 (7.5)	3895 (16.9)	
	31 to 35 years	44 (4.0)	1935 (8.4)	
	> 35 years	30 (2.7)	849 (3.7)	
	Missing	11	368	
Age at entry to motherhood (mean (SD))^a^		20.9 (5.0)	23.2 (5.6)	< 0.001

Missing data: Age at entry to motherhood (cohort: 11; comparison 368)

^a^ One-way analysis of variance tests were computed to compare differences in means between the cohort and matched comparison groups for continuous variables. Chi-squared analyses were used to compare all other differences for categorical variables in this table

However, the cohort mothers were significantly younger at age of entry to motherhood than the comparison group (mean age 20.9 and 23.2 years, respectively) (Table 1). Two-thirds (63.5%) were under 21 years old, 93.3% were aged 30 or under, and 2.7% were over 35 years at the time of birth, compared to 42.7%, 87.9% and 3.7% for these age bands in the comparison group.

### Measures of maternal health and well-being

**Fig. 2:**
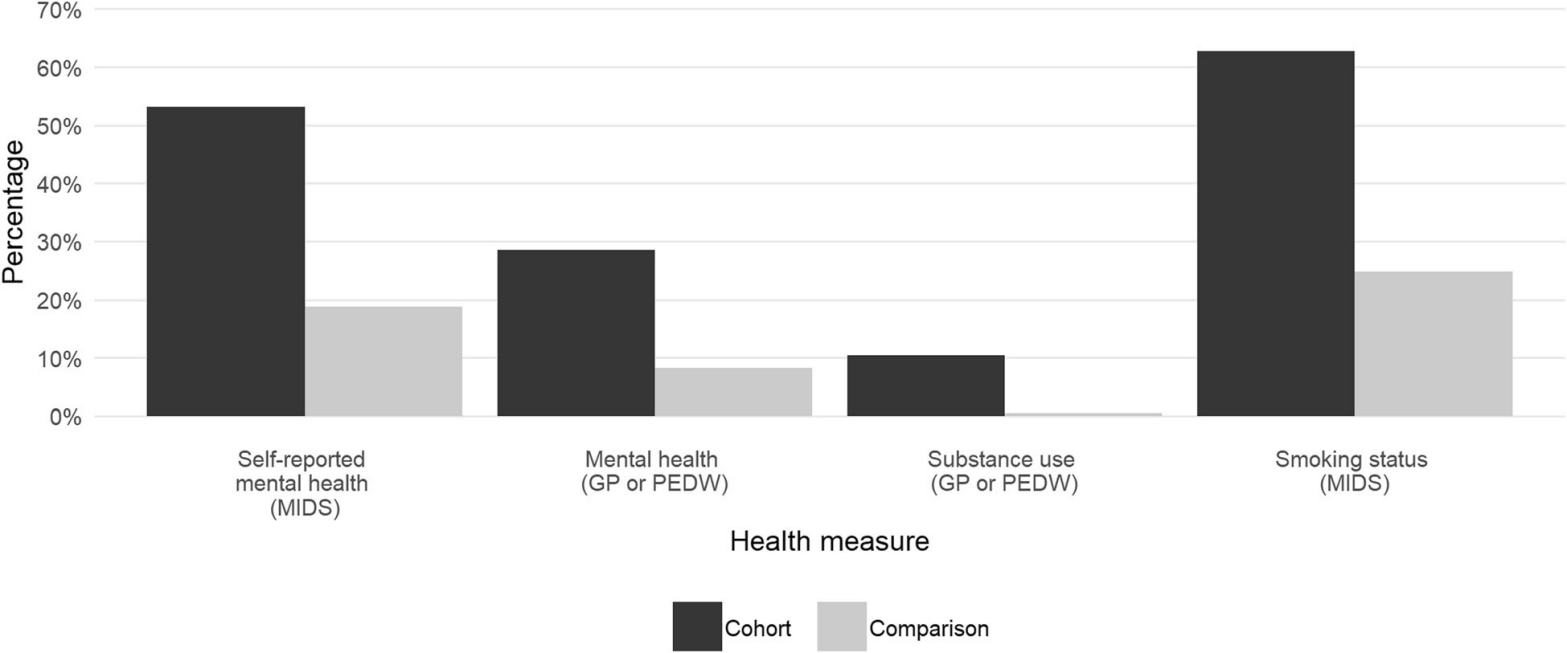
Prevalence of self-reported mental health, GP and hospital contacts or admissions for mental health and substance use, and self-reported smoking status, for the cohort (*n* = 1111) and comparison group (*n* = 23,414) during pregnancy

Half (53.2%) of the cohort, compared to 18.9% of the comparison group, self-reported having a mental health condition at their initial assessment, indicating the vulnerability of the cohort in this respect (Fig. 2, Table 2).

**Table 2:** Measures of mother health and wellbeing for the cohort (*n* = 1111) and comparison group (*n* = 23,414)

Variable	Level	Cohort n (%)	Comparison n (%)	***p***-value
Self-reported mental health	No	479 (46.8)	17,584 (81.1)	< 0.001
	Yes	544 (53.2)	4087 (18.9)	
Mental health-related contacts or admissions during pregnancy (within 9 months of birth)				
GP records	No	913 (82.2)	22,156 (94.6)	< 0.001
	Yes	198 (17.8)	1258 (5.4)	
PEDW records	No	950 (85.5)	22,574 (96.4)	< 0.001
	Yes	161 (14.5)	840 (3.6)	
GP or PEDW records	No	793 (71.4)	21,498 (91.8)	< 0.001
	Yes	318 (28.6)	1916 (8.2)	
Mental health-related contacts or admissions within 2 years prior to birth				
GP records	No	633 (57.0)	19,575 (83.6)	< 0.001
	Yes	478 (43.0)	3839 (16.4)	
PEDW records	No	882 (79.4)	22,265 (95.1)	< 0.001
	Yes	229 (20.6)	1149 (4.9)	
GP or PEDW records	No	536 (48.2)	18,989 (81.1)	< 0.001
	Yes	575 (51.8)	4425 (18.9)	
Substance use-related contacts or admissions during pregnancy (within 9 months of birth)				
GP records	No	1,026 (92.3)	23,331 (99.6)	< 0.001
	Yes	85 (7.7)	83 (0.4)	
PEDW records	No	1,050 (94.5)	23,358 (99.8)	< 0.001
	Yes	61 (5.5)	56 (0.2)	
GP or PEDW records	No	995 (89.6)	23,285 (99.4)	< 0.001
	Yes	116 (10.4)	129 (0.6)	
Substance use-related contacts or admissions within 2 years prior to birth				
GP records	No	959 (86.3)	23,190 (99.0)	< 0.001
	Yes	152 (13.7)	224 (1.0)	
PEDW records	No	999 (89.9)	23,195 (99.1)	< 0.001
	Yes	112 (10.1)	219 (0.9)	
GP or PEDW records	No	905 (81.5)	23,013 (98.3)	< 0.001
	Yes	206 (18.5)	401 (1.7)	
Body mass index (BMI)	Underweight	68 (6.9)	632 (3.0)	< 0.001
	Healthy weight	393 (39.9)	8844 (42.0)	
	Overweight	245 (24.9)	5773 (27.4)	
	Obese	221 (22.4)	4,847 (23.0)	
	Morbidly obese	58 (5.9)	961 (4.6)	
Smoker at initial assessment	No	396 (37.3)	17,028 (75.2)	
	Yes	667 (62.7)	5618 (24.8)	< 0.001
Smoker at birth	No	423 (40.4)	17,759 (78.4)	
	Yes	624 (59.6)	4879 (21.6)	< 0.001
Intention to breastfeed	No	685 (64.3)	10,584 (46.8)	
	Yes	380 (35.7)	12,037 (53.2)	< 0.001

Missing data (n): Self-reported mental health (cohort: 88; comparison 1743); BMI (cohort: 126; comparison: 2357); Smoking status at initial assessment (cohort: 48; comparison 768); Smoking status at birth (cohort: 64; comparison 776); Intention to breastfeed (cohort: 46; comparison 793)

Chi-squared analyses were used to compare differences between the cohort and matched comparison groups for all variables in this table

There was also a strong association between mental health related GP and hospital events during pregnancy, and within the 2 year period prior to the birth of the child involved in the current care proceedings (Table 2). During pregnancy, 17.8% of the cohort (compared to 5.4% of the comparison group) had at least one mental health related GP record, and 14.5% (compared to 3.6%) had at least one hospital event. Within the 2 year period prior to birth, 43.0% of the cohort (compared to 16.4%) had at least one mental health related GP record, and 20.6% (compared to 4.9%) had at least one hospital event. In total (using a combined measure of GP and hospital events for mental health), 28.6% of the cohort (compared to 8.2%) had a related record during pregnancy (Fig. 2), whilst 51.8% (compared to 18.9%) had a related record within the 2 year period prior to birth.

Results for substance use related contacts or admissions during pregnancy and within the 2 year period prior to the birth of the child involved in the current care proceedings showed similar disparity (Table 2). During pregnancy, 7.7% of the cohort had at least one related GP record and 5.5% had at least one hospital event, compared to < 0.5% of the comparison group for each of these measures. Within the 2 year period prior to birth, 13.7% of the cohort had at least one related GP record and 10.1% had at least one hospital event, compared to 1.0% of the comparison group for each of these measures. In total (using a combined measure of GP and hospital records for substance use), 10.4% of the cohort (compared to 0.6% the comparison group) had a related record during pregnancy (Fig. 2), whilst 18.5% (compared to 1.7%) had a related record within the 2 year period prior to birth. Thus, known substance use was far higher in the cohort than in the comparison group.

A significant association was found between mental health and substance use contact or admissions during pregnancy (*p* = 0.01) and 2 years prior to birth (*p* < 0.001) for the cohort (data not shown in table); of those with a mental health contact or admissions during pregnancy (*n* = 318) or 2 years prior to birth (*n* = 575), 14.2% and 23.8%, respectively, also had records for substance use. Incidentally, these two variables were also correlated in the comparison group.

In the cohort, 6.9% of the mothers were underweight at initial assessment (Table 2). Two-fifths (39.9%) had a healthy weight, a quarter (24.9%) were overweight (BMI 25–29.9), 22.4% were obese (BMI 30–39.9), and 5.9% were morbidly obese (BMI ≥ 40). Within the comparison group, fewer were underweight (3.0%) and morbidly obese (4.6%) but, otherwise the prevalence of women with healthy weights, overweight and obesity was largely similar.

Overall, 62.7% of the mothers in the cohort were smokers at initial assessment (Figure 2) and 59.6% smoked at childbirth (Table 2). This compares to 24.8 and 21.6% at each time point, respectively, of the mothers in the comparison group.

Fewer cohort mothers intended to breastfeed their babies – at around a third (35.7%) - compared to more than a half (53.2%) of mothers in the comparison group.

The cohort and comparison groups were significantly different for all of these measures of maternal health and wellbeing.

### Maternal reproductive history and interaction with midwifery services

At the time of their initial assessment, the mothers in the cohort reported significantly more previous pregnancies, with the range being one to eight. This was the first pregnancy for 25.5% of mothers and 37.6% reported four or more pregnancies (compared to 15.5% the comparison group) (Table 3).

**Table 3:** Maternal reproductive history and interaction with midwifery services for the cohort (*n* = 1111) and comparison group (*n* = 23,414)

Variable	Level	Cohort n (%)	Comparison n (%)	***p***-value
Gravida	1	261 (25.5)	9237 (43.3)	< 0.001
	2	206 (20.1)	5665 (26.5)	
	3	172 (16.8)	3147 (14.7)	
	> = 4	385 (37.6)	3307 (15.5)	
Parity	1	309 (30.8)	10,747 (51.1)	< 0.001
	2	232 (23.1)	6035 (28.7)	
	> = 3	463 (46.1)	4257 (20.2)	
Gestational age at initial assessment	1st trimester	659 (63.5)	18,506 (84.4)	< 0.001
	2nd trimester	311 (30.0)	2757 (12.6)	
	3rd trimester	68 (6.6)	654 (3.0)	
Gestational age at initial assessment (mean (SD))		13.3 (7.5)	10.5 (5.5)	< 0.001

Missing data (n): Gravida (cohort: 87; comparison 2058); Parity (cohort: 107; comparison: 2357); Gestational age at initial assessment (cohort: 73; comparison: 1497)

Chi-squared analyses were used to compare differences between the cohort and matched comparison groups for all variables in this table

It was the first live birth for 30.8% of the women in the cohort, second live birth for 23.1% and for the remaining 46.1% it was at least their third live birth (Table 3). The comparison group reported significantly lower parity; for example, for 51.1% it was their first live birth.

There was a significant association for gestational age at initial assessment, with cohort mothers tending to interact with maternity services later - the mean time was 13.3 and 10.5 weeks for the cohort and comparison mothers, respectively (Table 3); however, two-thirds (63.5%) of the cohorts mothers still had a timely initial assessment (within the first trimester of pregnancy), compared to 84.4% of the comparison group. More cohort mothers had a late initial assessment (during the third trimester) (6.6% compared to 3.0%).

### Immediate pregnancy and birth outcomes

The majority (> = 99.5%) of women in both the cohort and the comparison group had their baby in a hospital setting (Table 4).

**Table 4:** Immediate pregnancy and birth outcomes for the cohort (*n* = 1111) and comparison group (*n* = 23,414)

Variable	Level	Cohort n (%)	Comparison n (%)	***p***-value
Place of birth	Hospital	1105 (99.5)	23,344 (99.7)	0.443
	Non-Hospital	5 (0.5)	65 (0.3)	
Mode of birth	Spontaneous/unassisted	772 (69.7)	15,371 (65.8)	0.002
	Emergency C-Section	137 (12.4)	3210 (13.7)	
	Elective C-Section	114 (10.3)	2204 (9.4)	
	Forceps	58 (5.2)	1812 (7.8)	
	Ventouse	26 (2.3)	777 (3.3)	
Gestational age at onset of labour (mean (SD))^a^		38.4 (2.4)	39.1 (1.9)	< 0.001
Gestational age at onset of labour	Premature	153 (14.2)	1550 (6.7)	< 0.001
	Full term	926 (85.8)	21,429 (93.3)	
Birthweight for full-term babies	< 2500 g	74 (8.0)	607 (2.8)	< 0.001
	2500 g-3999 g	792 (85.2)	18,509 (86.2)	
	> = 4000 g	63 (6.8)	2346 (10.9)	
Birthweight for preterm babies (mean (SD))*		2,259.2 (685.7)	2,309.5 (718.7)	0.41
Apgar score	< 7	37 (3.4)	442 (1.9)	0.001
	> = 7	1043 (96.6)	22,618 (98.1)	

Missing data (n): Place of birth (cohort: 1; comparison: 5); Mode of birth (cohort: 4; comparison: 40); Gestational age at onset of labour (cohort: 32; comparison: 435); Birthweight for full-term babies (cohort: 2; comparison: 33); Birthweight for preterm babies (cohort: 2; comparison: 14); Apgar score (cohort: 31; comparison: 354)

^a^ One-way analysis of variance tests were computed to compare differences in means between the cohort and matched comparison groups. Chi-squared analyses were used to compare all other differences in this table

Over two-thirds (69.7%) of cohort births were vaginal (unassisted), with nearly a quarter (22.7%) of women delivering their baby via caesarean section (10.3% elective and 12.4% emergency) (Table 4). The remaining 7.5% of women had instrumental deliveries (forceps cephalic deliveries and ventouse (vacuum) deliveries). Mode of birth was similar for the comparison group (significant differences not observed).

A significantly greater proportion (14.2%) of the cohort had a preterm birth (< 37 weeks) - more than double the rate evident in our comparison group (6.7%).

The majority of babies were born full-term (≥ 37 weeks) and had birthweights within the ‘healthy weight’ range (2500 g-3999 g) in both the cohort and comparison group (85.2% and 86.2%, respectively). However, there were three times the proportion of babies with a low birth weight (< 2500 g) in the cohort (8.0%) than in the comparison group (2.8%). This was offset by a larger proportion of babies born weighing > = 4000 g in the comparison group (6.8% cohort, 10.9% comparison group). Birthweight categories of babies born preterm were not examined due to small cell sizes; however, overall, the mean birthweight of preterm babies was not significantly different for cohort and comparison group mothers (2259 g compared with 2309 g).

Although not significantly different, a slightly greater proportion of babies born to cohort mothers had Apgar scores below 7 (3.4% compared to 1.9%). While this represents a significant risk by nearly two-fold for the cohort, it still remains that nearly all babies in each group have a score 7 or above - a sign of good physical condition at birth.

## Discussion

### Summary of main findings

Mothers who experienced care proceedings before their child was 1 year of age showed heightened levels of need and/or vulnerability across multiple dimensions, when compared to other pregnant women in the matched comparison group. They were more likely to be younger at first motherhood, to self-report or have a health service contact/admission for mental health problems or substance use (drug and alcohol related), and to be smokers. Fewer intended to breastfeed their babies.

Almost two-thirds of mothers interacted with antenatal services at a timely point in pregnancy. A greater proportion of our cohort had preterm births, and a higher proportion of babies born full-term had low birth weights, however the vast majority of babies were born in a healthy condition according to their Apgar measure.

### Study strengths and limitations

This is the first time that population-level public family law records have been linked to maternity and other health data sources in Wales, enabled through the SAIL Databank. Studies based on administrative data are however necessarily limited by the scope and quality of available data, which is collected primarily for administrative rather than research purposes. Limitations of the Cafcass Cymru and MIDS datasets have previously been described [18, 29], but the SAIL Databank continues to work closely with data providers/owners to improve coverage, quality and quantity of its data sources. We acknowledge the possibility of some selection bias, which can occur if the records of certain subgroups of individuals have different linkage rates to other groups [30]; however, we do know that 90.9% of all Cafcass Cymru records relating to the mother for s.31 care proceedings between 2015 and 2018 were successfully matched in SAIL, enabling ALFs to be used to link to MIDS records and other study data sources. A further 10% of mothers were lost due to the study inclusion criteria, although we have described demographics of those who could not be retained in the final sample.

This study only reports on substance use and mental health problems during pregnancy and 2 years prior to birth that are both known to the healthcare practitioners and coded into patient records within the study period; as a result we cannot estimate or report on undiagnosed or pre-existing problems. Further, a small proportion of mothers may also have been registered at GP practices which do not supply their data to the SAIL Databank. Prevalences are therefore only for clinical presentation and are expected to be an underestimate of the true numbers of women with these problems and behaviours.

This descriptive study starts to build a picture of the health of mothers and babies involved in care proceedings but, as so little is known about the characteristics of this population, further research is required. This should continue to understand ‘who is coming to court’, reasons for this and what their needs and vulnerabilities are – such as more in-depth exploration of specific types of mental health problems, and individual alcohol and drug problems. Associations between poor maternal health and health-related habits and child health and development should also be explored. The longitudinal nature of EHRs within SAIL permits health service utilisation, illnesses and comorbidities during the postnatal period and further into child- and motherhood to be analysed.

### Comparison of research findings with previous literature

Previous research has also reported a clear association between deprivation and involvement in the family justice system and/or children’s entry to care [18, 31–33]; reasons for this may be multifactorial, including more women who have prior involvement with social services residing in these areas or, possibly, fewer support services being available for mothers at risk.

Population-level inequalities in some of the health indicators identified in this study, such as smoking and breastfeeding, have been established previously [34–37]; however, greater vulnerability in the cohort, compared to the comparison group, was still present despite using a matched-case-comparison study design.

Vulnerabilities identified, such as younger age at birth of first child, mental health problems and patterns of substance use have previously been found to be related to care proceedings and child maltreatment [38–40]. Other studies looking at mothers who have lost the care of their infant also report greater drug [41, 42] and alcohol use during pregnancy [43].

The perinatal period is critical for the healthy development of infants [44]. Smoking, alcohol consumption, drug use and mental health problems can have an adverse impact on the development of the fetus and longer-term child outcomes [45, 46]. This study identified more preterm births and babies with low birth weights in the cohort than in the comparison group, but the majority of births showed signs of good health at birth; further research is required to examine associations between maternal health and health-related habits, and these immediate birth and pregnancy outcomes as described above. These further analyses should consider confounding factors, such as smoking status during pregnancy, on birth outcomes.

Existing evidence suggests that women involved in care proceedings have fewer prenatal visits [47] out of fear that children’s services will remove the baby at birth, however, largely, avoidance of engagement with antenatal services was not common for this particular population of mothers in this study, given that almost two-thirds of them had booked an initial assessment by the first trimester of pregnancy.

### Recommendations for policy and practice

Concerns about how pregnant mothers who are at risk of involvement in the family courts might be helped, are not specific to Wales or the UK. In a number of international contexts, there is growing interest in pre-birth assessment and early intervention for ‘high risk’ mothers [5, 48]. However, studies that have focused specifically on the population of women whose infants were subject to care proceedings are very few in number [4, 49]. This study demonstrates the potential of record linkage to throw light on questions about antenatal engagement and women’s health vulnerabilities, specific to women appearing in family court proceedings, who might otherwise be hard to reach.

Maternity staff in Wales are directed to ensuring that pregnant women and their families receive personalised and timely support with safe, clinically effective, care [50]. Since the Marmot Review [44] there has been a specific focus on the first 1000 days, from conception to the age of two, to improve outcomes and reduce inequalities during this critical period. Whilst this is supported by Welsh Government and the national public health agency [51, 52], and a delivery plan has been put in place to improve, for example, perinatal mental health services [53], a review of provision suggests that women cannot consistently access support services as specialist teams are stretched beyond capacity [54].

Pregnant women who are at risk of becoming involved in care proceedings require more intensive, earlier, engagement with maternity and other support services to prevent recurrent care proceedings [55, 56]. Pre-birth assessments are also required to manage the rights and needs of families, and to prompt or evidence change in parenting capacity [2]. New and innovative approaches to practice can also succeed with vulnerable parents, where the local authority standard casework model has struggled to turn lives around. Interventions aimed at offering greater support for parents who have had children removed previously can help parents around issues such as contraception, housing, substance misuse and mental health. “Reflect” is one such service in Wales [57]. The “Baby and Me” service in Newport, Wales, which is delivered by Barnardos [58], is also another example of an intensive targeted service. This is a service resulting from collaboration between the local authority and the third sector, working together to design services that meet the needs of parents requiring higher levels of support. Through involvement with the service, a number of parents who may otherwise have lost their children to public care or adoption, have managed to keep their babies. For parents who have not been able to make sufficient change – “Baby and Me” has still been a positive experience, enabling parents to accept and understand the reasons why children were removed.

Professionals delivering such support understand that these parents may have significant problems and needs, and difficulties in engaging with the structure and formal appointment systems of mainstream services. They work intensively with parents, and see their own relationships with families as the vehicle for change, reducing common difficulties and – importantly - serving as a bridge to more specialist treatments where these are needed.

Such preventative services also need to be responsive to the needs of mothers not already known to services, including first-time mothers and other vulnerable pregnant women – including those who have been in care themselves. Interventions targeting these groups may require additional and more rapid strategies, such as assertive outreach, flexible appointment times and accessible clear information [38]. Equally, there is a pressing need to examine the skill base of universal services, given current and likely funding constraints. Midwives and health visitors in mainstream services are well placed to respond to lower level mental health needs and other health vulnerabilities, given evidence in this study of the timing of women’s first engagement with antenatal services.

## Conclusion

Health professionals, social workers and family support workers have a key role to play in identifying vulnerable women and ensuring that they receive appropriate support. Enabling the early provision of support services is essential for all women, especially those who have previously been involved with social services, if we are to give every child the best start in life [44] and reduce the numbers of infants subject to child protection concerns and coming before the family courts in care proceedings.

The prenatal period presents a critical opportunity for services to engage with women experiencing difficulties with substance use, mental health or other adverse circumstances. Policy and practice colleagues require a clear picture of families involved in the family justice system in order to tailor effective and preventative services, and specialist care, to make the best evidence-informed decisions.

## Supplementary Information

**Additional file 1 **: **Table SM1.** Unadjusted odds ratios (OR) with 99% Confidence Intervals (CIs) for all measures in relation to being in the cohort over the comparison group.

## Data Availability

The data used in this study is available from the Secure Anonymised Information Linkage (SAIL) Databank at Swansea University, Swansea, UK, which is part of the national e-health records research infrastructure for Wales. Those wishing to access data should follow the application process guidelines available at: www.saildatabank.com/application-process

## Abbreviations

EHRs: Electronic health records
WIMD: Welsh Index of Multiple Deprivation
MIDS: Maternity Indicators Dataset
PEDW: Patient Episode Database for Wales
WLGP: Welsh Longitudinal General Practice data
IGRP: SAIL Information Governance Review Panel
SAIL Databank: Secure Anonymised Information Linkage
GP: General Practice

## References

[1] Alrouh B, Broadhurst K, Cusworth L, Griffiths L, Johnson RD, Akbari A (2019). Born into care Wales: newborns and infants in care proceedings in Wales.

[2] Mason C, Robertson L, Broadhurst K (2019). Pre-birth Assessment and Infant Removal at Birth: Experiences and Challenges - A literature review.

[3] Grant T, Graham JC, Ernst CC, Peavy KM, Brown NN (2014). Improving pregnancy outcomes among high-risk mothers who abuse alcohol and drugs: factors associated with subsequent exposed births. Child Youth Serv Rev.

[4] Grant T, Huggins J, Graham JC, Ernst C, Whitney N, Wilson D (2011). Maternal substance abuse and disrupted parenting: distinguishing mothers who keep their children from those who do not. Child Youth Serv Rev.

[5] Taplin S (2017). Prenatal reporting to child protection: characteristics and service responses in one Australian jurisdiction. Child Abuse Negl.

[6] Care Crisis Review options for change. 2018. Available from: www.nuffieldfoundation.org (Accessed 22 Sept 2020).

[7] Wall-Wieler E, Kenny K, Lee J, Thiessen K, Morris M, Roos LL (2019). Prenatal care among mothers involved with child protection services in Manitoba: a retrospective cohort study. CMAJ..

[8] McKetta S, Keyes KM (2019). Heavy and binge alcohol drinking and parenting status in the United States from 2006 to 2018: an analysis of nationally representative cross-sectional surveys. PLoS Med.

[9] Vesga-López O, Blanco C, Keyes K, Olfson M, Grant BF, Hasin DS (2008). Psychiatric disorders in pregnant and postpartum women in the United States. Arch Gen Psychiatry.

[10] Jordan S, Davies GI, Thayer DS, Tucker D, Humphreys I (2019). Antidepressant prescriptions, discontinuation, depression and perinatal outcomes, including breastfeeding: a population cohort analysis. PLoS One.

[11] Broadhurst K, Budd T, Williams T. The Nuffield family justice Observatory for England and Wales: making it happen. London; 2018.

[12] Lyons RA, Jones KH, John G, Brooks CJ, Verplancke J-P, Ford DV (2009). The SAIL databank: linking multiple health and social care datasets. BMC Med Inform Decis Mak.

[13] Ford DV, Jones KH, Verplancke J-P, Lyons RA, John G, Brown G (2009). The SAIL databank: building a national architecture for e-health research and evaluation. BMC Health Serv Res.

[14] Jones KH, Laurie G, Stevens L, Dobbs C, Ford DV, Lea N (2017). The other side of the coin: harm due to the non-use of health-related data. Int J Med Inform.

[15] Jones KH, Ford DV, Thompson S, Lyons R. A Profile of the SAIL Databank on the UK Secure Research Platform. Int J Popul Data Sci. 2019;4(2):1134. 10.23889/ijpds.v4i2.1134 PMC8142954 34095541

[16] Bedston S, Pearson RA, Jay MA, Broadhurst K, Gilbert R, Wijlaars L. Data Resource: Children and Family Court Advisory and Support Service (Cafcass) public family law administrative records in England. Int J Popul Data Sci. 2020;5(1):1159. 10.23889/ijpds.v5i1.1159 PMC7482375 34232967

[17] Broadhurst K, Shaw M, Kershaw S, Harwin J, Alrouh B, Mason C (2015). Vulnerable birth mothers and repeat losses of infants to public care: is targeted reproductive health care ethically defensible?. J Soc Welf Fam Law.

[18] Johnson RD, Ford DV, Broadhurst K, Cusworth L, Jones KH, Akbari A (2020). Data resource: population level family justice administrative data with opportunities for data linkage. Int J Popul Data Sci.

[19] Maternity Indicators Data Set. Available from: http://www.datadictionary.wales.nhs.uk/index.html#!WordDocuments/maternityindicatorsdatasetmids.htm (Accessed 04 Nov 2020).

[20] Public Health Wales Observatory | Patient Episode Database for Wales (PEDW). Available from: http://www.publichealthwalesobservatory.wales.nhs.uk/pedw/ (Accessed 14 May 2020).

[21] ICD-10 Version:2016. Available from: https://icd.who.int/browse10/2016/en (Accessed 14 May 2020).

[22] Office for National Statistics. 2011 Census: population and household estimates for small areas in England and Wales, 2011. Available from: https://www.ons.gov.uk/peoplepopulationandcommunity/populationandmigration/populationestimates/bulletins/2011censuspopulationandhouseholdestimatesforsmallareasinenglandandwales/2012-11-23 (Accessed 24 Mar 2020).

[23] The Platform - Adolescent Mental Health Data Platform. Available from: https://adolescentmentalhealth.uk/Platform (Accessed 28 Feb 2020).

[24] John A, Marchant AL, Fone DL, McGregor JI, Dennis MS, Tan JOA (2016). Recent trends in primary-care antidepressant prescribing to children and young people: an e-cohort study. Psychol Med.

[25] John A, McGregor J, Fone D, Dunstan F, Cornish R, Lyons RA (2016). Case-finding for common mental disorders of anxiety and depression in primary care: an external validation of routinely collected data. BMC Med Inform Decis Mak..

[26] John A, McGregor J, Jones I, Lee SC, Walters JTR, Owen MJ (2018). Premature mortality among people with severe mental illness - new evidence from linked primary care data. Schizophr Res.

[27] Demmler JC, Brophy ST, Marchant A, John A, Tan JOA (2020). Shining the light on eating disorders, incidence, prognosis and profiling of patients in primary and secondary care: national data linkage study. Br J Psychiatry.

[28] R: The R Project for Statistical Computing. Available from: https://www.r-project.org/ (Accessed 5 June 2020).

[29] Knowledge and Analytical Services, Welsh Government (2019). Quality Report: Maternity and Birth Statistics.

[30] Bohensky M. Bias in data linkage studies. In: Harron K, Goldstein H, Dibben C. Methodological Developments in Data Linkage. 2016. p. 63–82.

[31] Bywaters P, Brady G, Sparks T, Bos E (2016). Child welfare inequalities: new evidence, further questions. Child Fam Soc Work.

[32] Harwin J, Alrouh B. New entrants and repeat children: continuity and change in care demand over time. Fam Law. 2017:404–11.

[33] Elliott M (2019). Child welfare inequalities in a time of rising numbers of children entering out-of-home care. Br J Soc Work.

[34] Fone D, Dunstan F, Lloyd K, Williams G, Watkins J, Palmer S (2007). Does social cohesion modify the association between area income deprivation and mental health? A multilevel analysis. Int J Epidemiol.

[35] Goodwin RD, Cheslack-Postava K, Nelson DB, Smith PH, Hasin DS, Janevic T (2017). Serious psychological distress and smoking during pregnancy in the United States: 2008-2014. Nicotine Tob Res.

[36] Peregrino AB, Watt RG, Heilmann A, Jivraj S (2018). Breastfeeding practices in the United Kingdom: is the neighbourhood context important?. Matern Child Nutr.

[37] Riaz M, Lewis S, Naughton F, Ussher M (2018). Predictors of smoking cessation during pregnancy: a systematic review and meta-analysis. Addiction.

[38] Canfield M, Radcliffe P, Marlow S, Boreham M, Gilchrist G (2017). Maternal substance use and child protection: a rapid evidence assessment of factors associated with loss of child care. Child Abus Negl.

[39] Roscoe JN, Lery B, Chambers JE (2018). Understanding child protection decisions involving parents with mental illness and substance abuse. Child Abus Negl..

[40] Baldwin H, Biehal N, Allgar V, Cusworth L, Pickett K (2020). Antenatal risk factors for child maltreatment: linkage of data from a birth cohort study to child welfare records. Child Abuse Negl.

[41] Simmat-Durand L, Lejeune C (2012). Polydrug use during pregnancy and neonatal outcome: data from a ten-year retrospective French study. J Neonatal Nurs.

[42] Tsantefski M, Humphreys C, Jackson AC (2014). Infant risk and safety in the context of maternal substance use. Child Youth Serv Rev.

[43] Sarkola T, Kahila H, Gissler M, Halmesmäki E (2007). Risk factors for out-of-home custody child care among families with alcohol and substance abuse problems. Acta Paediatr.

[44] Marmot M. Fair Society, Healthy Lives. The Marmot Review: Strategic review of health inequalities in England post-2010. 2010.

[45] Zhao L, McCauley K, Sheeran L (2017). The interaction of pregnancy, substance use and mental illness on birthing outcomes in Australia. Midwifery..

[46] Mamluk L, Edwards HB, Savović J, Leach V, Jones T, Moore THM (2017). Low alcohol consumption and pregnancy and childhood outcomes: Time to change guidelines indicating apparently “safe” levels of alcohol during pregnancy? A systematic review and meta-analyses. BMJ Open.

[47] Minnes S, Singer LT, Humphrey-Wall R, Satayathum S (2008). Psychosocial and behavioral factors related to the post-partum placements of infants born to cocaine-using women. Child Abuse Negl.

[48] Taylor L, Hutchinson D, Rapee R, Burns L, Stephens C, Haber PS. Clinical features and correlates of outcomes for high-risk, marginalized mothers and newborn infants engaged with a specialist perinatal and family drug health service. Obstet Gynecol Int. 2012;867265. 10.1155/2012/867265. 10.1155/2012/867265 PMC3512313 23227054

[49] Taplin S, Mattick RP (2014). Supervised contact visits: results from a study of women in drug treatment with children in care. Child Youth Serv Rev.

[50] Welsh Government (2019). Maternity Care in Wales-a 5 year vision for the future.

[51] Welsh Government (2019). A healthier Wales: our plan for health and social care.

[52] Cymru Well Wales: The First 1000 Days (F1000D). Available from: http://www.wales.nhs.uk/sitesplus/888/page/88523. (Accessed 29 Sept 2020).

[53] Welsh Government (2019). Together for mental health delivery plan: 2019-22.

[54] Witcombe-Hayes S, Cymru N, Wales /, Professor W, Jones I, Jones S, et al. From bumps to babies: perinatal mental health care in Wales - full report (English). 2018. Cardiff: NSPCC, National Centre for Mental Health, Mind Cymru, Mental Health Foundation, Maternal Mental Health Everyone’s Business.

[55] Alrouh B, Broadhurst K, Cusworth L (2020). Women in recurrent care proceedings in Wales: a first benchmarking report.

[56] Griffiths LJ, Johnson RD, Broadhurst K, Cusworth L, Bedston S, Jones KH (2020). Born into care: one thousand mothers in care proceedings in Wales.

[57] Barnardo’s Reflect. Available from: https://www.exchangewales.org/barnardos-reflect/ (Accessed 04 Nov 2020).

[58] Believe in children | Children’s charity | Barnardo’s. Available from: https://www.barnardos.org.uk/ (Accessed 22 Sept 2020).

