# Supplementary material for: Maternal health, pregnancy and birth outcomes for women involved in care proceedings in Wales: a linked data study
Source: BMC Pregnancy Childbirth. 2020 Nov 16;20:697. doi: 10.1186/s12884-020-03370-4 (PMC7667744; doi:10.1186/s12884-020-03370-4)
Supplement: Supplementary file 1 — Additional file 1 : Table SM1. Unadjusted odds ratios (OR) with 99% Confidence Intervals (CIs) for all measures in relation to being in the cohort over the comparison group. [file 12884_2020_3370_MOESM1_ESM.docx]

**Maternal health, pregnancy and birth outcomes for women involved in care proceedings in Wales: a linked data study**

**Supplementary Material**

**Table SM1:** Unadjusted odds ratios (OR) with 99% Confidence Intervals (CIs) for all measures in relation to being in the cohort over the comparison group.

| **Variable** | **Term** | **OR (99% CI)** |
| --- | --- | --- |
| Grand Odds Ratio |  | 0.047 (0.047, 0.048) |
| Age at entry to motherhood | <21 years | 0.071 (0.062, 0.081) |
|  | 21 to 25 years | 0.038 (0.030, 0.046) |
|  | 26 to 30 years | 0.021 (0.014, 0.030) |
|  | 31 to 35 years | 0.023 (0.013, 0.036) |
|  | >35 years | 0.035 (0.018, 0.062) |
| Self-reported mental health | No | 0.027 (0.023, 0.032) |
|  | Yes | 0.133 (0.114, 0.154) |
| MH_GP_9 months | No | 0.041 (0.037, 0.046) |
|  | Yes | 0.157 (0.121, 0.201) |
| MH_PEDW_9 months | No | 0.042 (0.038, 0.047) |
|  | Yes | 0.192 (0.143, 0.252) |
| MH_GP/PEDW_9 months | No | 0.037 (0.033, 0.041) |
|  | Yes | 0.166 (0.135, 0.202) |
| MH_GP_2 years | No | 0.032 (0.028, 0.037) |
|  | Yes | 0.125 (0.106, 0.146) |
| MH_PEDW_2 years | No | 0.040 (0.035, 0.044) |
|  | Yes | 0.199 (0.156, 0.251) |
| MH_GP/PEDW_2 years | No | 0.028 (0.024, 0.032) |
|  | Yes | 0.130 (0.112, 0.150) |
| SU_GP_9 months | No | 0.044 (0.040, 0.049) |
|  | Yes | 1.024 (0.615, 1.707) |
| SU_PEDW_9 months | No | 0.045 (0.040, 0.050) |
|  | Yes | 1.089 (0.591, 2.018) |
| SU_GP/PEDW_9 months | No | 0.043 (0.038, 0.047) |
|  | Yes | 0.899 (0.588, 1.370) |
| SU_GP_2 years | No | 0.041 (0.037, 0.046) |
|  | Yes | 0.679 (0.478, 0.956) |
| SU_PEDW_2 years | No | 0.043 (0.039, 0.048) |
|  | Yes | 0.511 (0.346, 0.745) |
| SU_GP/PEDW_2 years | No | 0.039 (0.035, 0.044) |
|  | Yes | 0.514 (0.386, 0.679) |
| Body mass index (BMI) | Underweight | 0.108 (0.069, 0.160) |
|  | Healthy Weight | 0.044 (0.037, 0.052) |
|  | Overweight | 0.042 (0.034, 0.052) |
|  | Obese | 0.046 (0.036, 0.057) |
|  | Morbidly Obese | 0.060 (0.037, 0.092) |
| Smoker at initial assessment | Smoker | 0.119 (0.104, 0.136) |
|  | Non-smoker | 0.023 (0.020, 0.027) |
| Smoker at birth | Smoker | 0.128 (0.111, 0.147) |
|  | Non-smoker | 0.024 (0.020, 0.028) |
| Intention to breastfeed | Yes | 0.032 (0.026, 0.037) |
|  | No | 0.065 (0.057, 0.074) |
| Gravida | 1 | 0.028 (0.023, 0.035) |
|  | 2 | 0.036 (0.029, 0.046) |
|  | 3 | 0.055 (0.042, 0.070) |
|  | >=4 | 0.116 (0.097, 0.138) |
| Parity | 1 | 0.029 (0.024, 0.035) |
|  | 2 | 0.038 (0.031, 0.048) |
|  | 3 | 0.109 (0.092, 0.127) |
| Gestational age at initial assessment | 1st trimester | 0.036 (0.031, 0.040) |
|  | 2nd trimester | 0.113 (0.092, 0.137) |
|  | 3rd trimester | 0.104 (0.067, 0.155) |
| Place of birth | Hospital | 0.047 (0.043, 0.052) |
|  | Non-hospital | 0.077 (0.011, 0.274) |
| Mode of birth | Spontaneous | 0.050 (0.044, 0.057) |
|  | Emergency C-Section | 0.043 (0.032, 0.056) |
|  | Elective C-Section | 0.052 (0.037, 0.070) |
|  | Forceps | 0.032 (0.020, 0.048) |
|  | Ventouse | 0.033 (0.016, 0.061) |
| Gestational age at onset of labour | Premature | 0.099 (0.074, 0.129) |
|  | Full term | 0.043 (0.039, 0.048) |
| Birthweight for full-term babies | <2500g | 0.122 (0.079, 0.179) |
|  | 2500g-2999g | 0.074 (0.060, 0.091) |
|  | 3000g-3499g | 0.040 (0.033, 0.048) |
|  | 3500g-3999g | 0.030 (0.023, 0.038) |
|  | >=4000g | 0.027 (0.017, 0.040) |
| Apgar score | <7 | 0.084 (0.045, 0.141) |
|  | >=7 | 0.046 (0.041, 0.051) |
